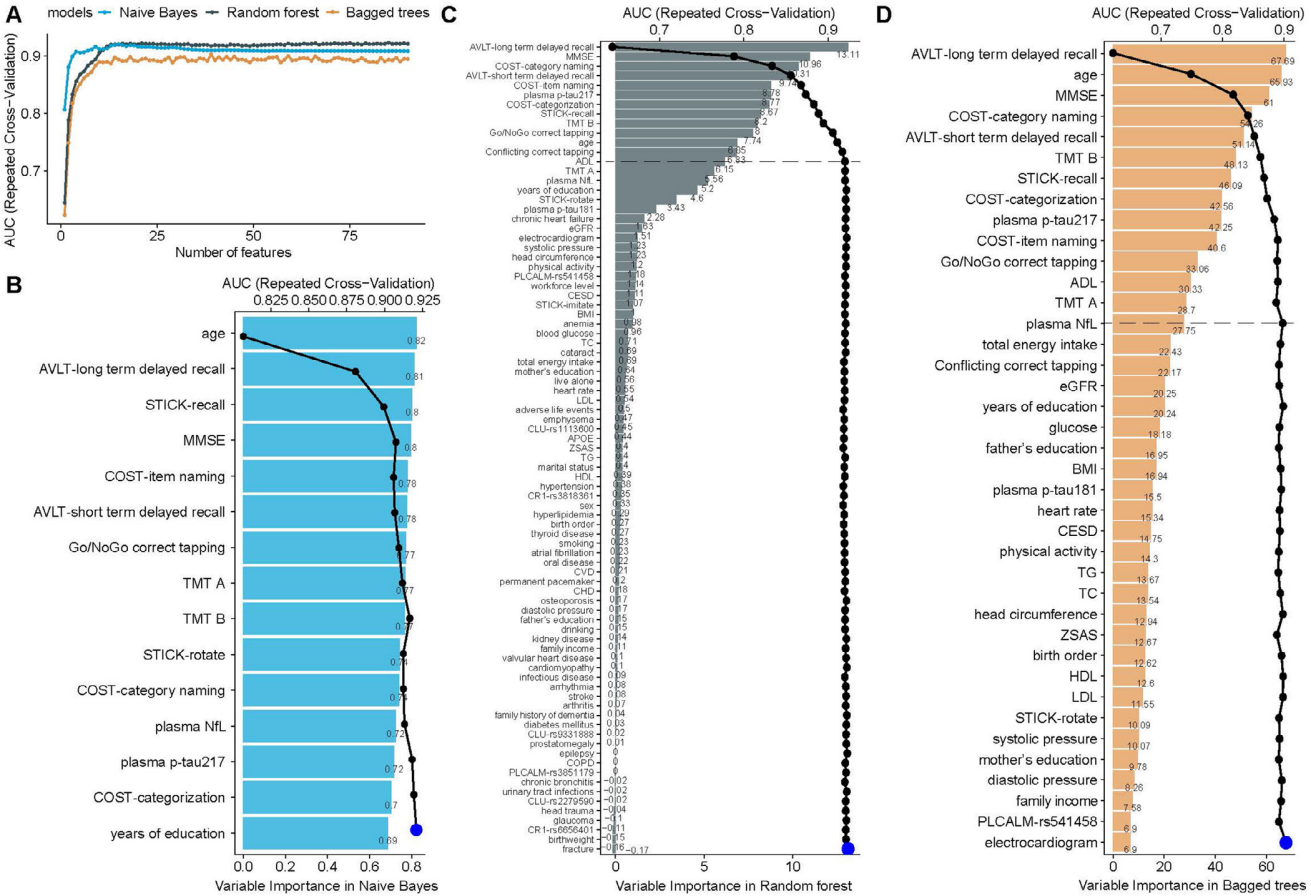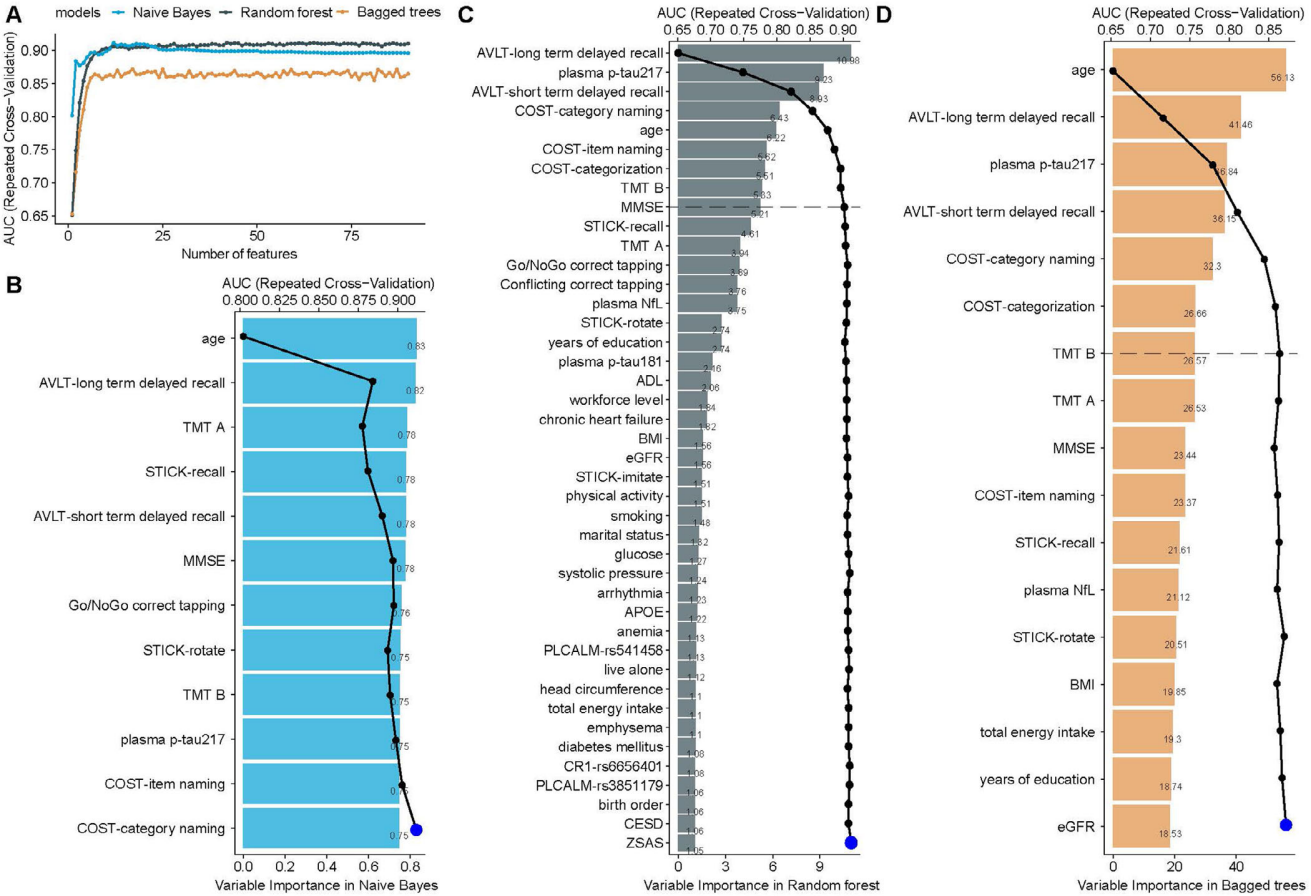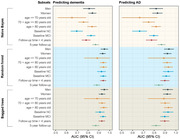# Significance of plasma biomarkers in predicting long‐term dementia risk in community dwellings: insights from machine learning approaches

**DOI:** 10.1002/alz.091342

**Published:** 2025-01-09

**Authors:** Zhenxu Xiao, Xiaowen Zhou, Qianhua Zhao, Yang Cao, Ding Ding

**Affiliations:** ^1^ Institute of Neurology, Huashan Hospital, Fudan University, Shanghai China; ^2^ National Center for Neurological Disorders, Huashan Hospital, Fudan University, Shanghai China; ^3^ National Clinical Research Center for Aging and Medicine, Huashan Hospital, Fudan University, Shanghai China; ^4^ MOE Frontiers Center for Brain Science, Fudan University, Shanghai China; ^5^ Clinical Epidemiology and Biostatistics, Department of Medical Sciences, Faculty of Medicine and Health, Örebro University, Örebro Sweden; ^6^ Unit of Integrative Epidemiology, Institute of Environmental Medicine, Karolinska Institute, Stockholm Sweden

## Abstract

**Background:**

The role of plasma biomarkers for predicting incident dementia in the general population is still undetermined.

**Method:**

A total of 1857 baseline non‐demented older adults with follow‐ups within 10 years were included from a community‐based cohort. Consensus diagnoses of dementia and Alzheimer’s disease (AD) were based on clinical criteria. The Recursive Feature Elimination algorithm was used to select the important features from 90 baseline candidate predictors for developing dementia and AD prediction models. Area Under the Receiver Operating Characteristic Curve (AUC) was used to evaluate models’ performance. Models were further validated in the testing sets and subsets.

**Result:**

During the follow‐up of 12,716 person‐years, 207 participants developed dementia (including 145 AD). The constructed Naive Bayes, Random Forest, and Bagged Trees models showed moderate‐to‐high AUCs predicting future dementia (AUCs = 0.700‐0.828) and AD (AUCs = 0.630‐0.844) in the testing sets. Age, neuropsychological tests, plasma p‐tau217, and neurofilament light chain (NfL) were the prioritized variables in all models. The models showed robustness in diverse subgroups.

**Conclusion:**

Plasma p‐tau217 and NfL in combination with age and cognitive performance showed important values in predicting incident dementia and AD among community older adults. In the resource‐limited settings, these variables may be convenient‐collected and cost‐effective to identify participants with high risk of dementia in the general population.